# Executive functioning, behavior, and white matter microstructure in the chronic phase after pediatric mild traumatic brain injury: results from the adolescent brain cognitive development study

**DOI:** 10.1017/S0033291724000229

**Published:** 2024-07

**Authors:** Anja K. Betz, Suheyla Cetin-Karayumak, Elena M. Bonke, Johanna Seitz-Holland, Fan Zhang, Steve Pieper, Lauren J. O'Donnell, Yorghos Tripodis, Yogesh Rathi, Martha E. Shenton, Inga K. Koerte

**Affiliations:** 1 cBRAIN, Department of Child and Adolescent Psychiatry, Psychosomatics and Psychotherapy, Ludwig-Maximilians-Universität, Munich, Germany; 2 Psychiatry Neuroimaging Laboratory, Brigham and Women's Hospital, Harvard Medical School, Boston, MA, USA; 3 Graduate School of Systemic Neurosciences, Ludwig-Maximilians-Universität, Munich, Germany; 4 Department of Psychiatry, Massachusetts General Hospital, Harvard Medical School, Boston, MA, USA; 5 Department of Radiology, Brigham and Women's Hospital, Harvard Medical School, Boston, MA, USA; 6 Isomics, Inc., Cambridge, MA, USA; 7 Department of Biostatistics, Boston University School of Public Health, Boston, MA, USA

**Keywords:** concussion, diffusion magnetic resonance imaging, long-term outcome, pediatric mild traumatic brain injury

## Abstract

**Background:**

Mild traumatic brain injury (mTBI) is common in children. Long-term cognitive and behavioral outcomes as well as underlying structural brain alterations following pediatric mTBI have yet to be determined. In addition, the effect of age-at-injury on long-term outcomes is largely unknown.

**Methods:**

Children with a history of mTBI (*n* = 406; *M*_age_ = 10 years, *SD*_age_ = 0.63 years) who participated in the *Adolescent Brain Cognitive Development* (ABCD) study were matched (1:2 ratio) with typically developing children (TDC; *n* = 812) and orthopedic injury (OI) controls (*n* = 812). Task-based executive functioning, parent-rated executive functioning and emotion-regulation, and self-reported impulsivity were assessed cross-sectionally. Regression models were used to examine the effect of mTBI on these domains. The effect of age-at-injury was assessed by comparing children with their first mTBI at either 0-3, 4-7, or 8-10 years to the respective matched TDC controls. Fractional anisotropy (FA) and mean diffusivity (MD), both MRI-based measures of white matter microstructure, were compared between children with mTBI and controls.

**Results:**

Children with a history of mTBI displayed higher parent-rated executive dysfunction, higher impulsivity, and poorer self-regulation compared to both control groups. At closer investigation, these differences to TDC were only present in one respective age-at-injury group. No alterations were found in task-based executive functioning or white matter microstructure.

**Conclusions:**

Findings suggest that everyday executive function, impulsivity, and emotion-regulation are affected years after pediatric mTBI. Outcomes were specific to the age at which the injury occurred, suggesting that functioning is differently affected by pediatric mTBI during vulnerable periods. Groups did not differ in white matter microstructure.

## Introduction

Mild traumatic brain injury (mTBI) is common and affects about 1 million children annually in the US alone (Mannix, O'Brien, & Meehan, 2013). While most children recover from acute symptoms within weeks following mTBI, about 30% experience prolonged post-concussive symptoms months later (Babcock et al., 2013). Moreover, the developmental gap between children with TBI and typically developing children (TDC) may widen with time because complex skills fail to develop properly (Babikian, Merkley, Savage, Giza, & Levin, 2015). To date, however, most studies of pediatric mTBI focus primarily on the first few months following injury (Goh et al., 2021; Schmidt et al., 2018). Research is thus needed to investigate long-term outcomes following pediatric mTBI and the underlying pathophysiology to guide more targeted therapeutic interventions.

Executive functioning comprises a wide range of functions that relate to cognitive control, including inhibition, shifting between tasks, working memory, planning, and decision-making (Levin & Hanten, 2005). Their development is often tied to the maturation and myelination of the frontal cortex white matter, both of which continue into early adulthood (Lebel & Beaulieu, 2011). Due to the high prevalence of diffuse axonal injuries after TBI, the developing white matter microstructure and corresponding executive abilities may be particularly vulnerable to pediatric TBI (Pinto, Poretti, Meoded, Tekes, & Huisman, 2012). According to a recent meta-analysis, executive functions are, in fact, affected by pediatric mTBI with impairments that last for months to years after the injury (Goh et al., 2021). However, other studies report either no impairment in executive functioning (Maillard-Wermelinger et al., 2009) or initial difficulties that resolve within months post-injury (Chadwick et al., 2021). The inconsistent findings may be due to the different methods used to assess executive functioning. Task-based assessment and rating scales are often interpreted interchangeably, even though they may measure distinct functions in different contexts (Friedman & Banich, 2019). While task-based assessments of executive functioning are believed to isolate one cognitive process under standardized conditions, rating scales reflect executive functioning in everyday situations (Friedman & Banich, 2019; Lace et al., 2019). If and how pediatric mTBI affects these different aspects of executive functioning in the long-term remains to be determined.

Problems with executive functioning and, subsequently, behavioral control may be observed as impulsive behavior (Nigg, 2017). Whilst impulsivity has been tied to sports-related concussion in adolescents and young adults (Liebel, Edwards, & Broglio, 2021), it is rarely investigated in children. Doing so has great clinical relevance because higher impulsivity is associated with an increased risk of needing psychiatric care following pediatric mTBI (Saarinen et al., 2022).

Further, persistent emotional problems have frequently been reported following mTBI in children (Emery et al., 2016; Ewing-Cobbs et al., 2021; Gagner, Landry-Roy, Bernier, Gravel, & Beauchamp, 2018; Jones et al., 2021). In addition to simply *experiencing* negative emotions, emotional problems may be due to impairments in cognitive control and subsequent difficulties in the *regulation* of such emotions.

Importantly, investigating consequences of mTBI in children and adolescents also needs to consider effects of brain development (i.e., brain injury ‘hits a moving target’; Giza, Kolb, Harris, Asarnow, & Prins, 2009). A TBI may not only impact already established functions but may also influence the developmental trajectory of new functions. Children's cognitive and behavioral abilities are formed during different time-periods of development and at different paces. This means that outcomes following mTBI may depend on the age at which it occurs (Anderson et al., 2009; Serpa et al., 2021). Further, many additional factors influence brain development. To account for environmental factors (e.g., socio-economic status), children with mTBI need to be compared to closely matched controls. Additionally, research on behavioral effects of mTBI needs to consider pre-injury characteristics (e.g., higher impulsivity predisposing children for injuries) and general injury effects (e.g., emotional distress after injury). Therefore, children with a history of orthopedic injury (OI) constitute a valuable control group in addition to TDC (Babikian et al., 2011; Emery et al., 2016).

Finally, while long-term behavioral difficulties following pediatric mTBI have received increasing attention, research on their neural mechanisms remains sparse. Diffusion MR imaging (dMRI) has been proposed as a sensitive tool for detecting alterations in brain microstructure following mTBI (Königs et al., 2018; Shenton et al., 2012). In fact, one study reports initial indications of a developmental stall in white matter microstructure, with fractional anisotropy (FA) increasing over time in TDC but not in children with a complicated mild or moderate TBI (Bartnik-Olson et al., 2021). However, the literature on white matter microstructure in pediatric mTBI is inconsistent (Jain, Das, Agrawal, Babal, & Purohit, 2021) and, to date, it is largely unknown if alterations in white matter microstructure are present years after pediatric mTBI (Lindsey, Hodges, Greer, Wilde, & Merkley, 2021).

In this study, children with a history of mTBI were expected to exhibit worse task-based and parent-rated executive functioning, more impulsive behavior, and worse emotion-regulation when compared to TDC and OI controls based on the large and representative Adolescent Brain and Cognitive Development (ABCD) study. Further, we explore whether the age at first mTBI has an influence on the differences from controls. Finally, we investigate whether children with a history of mTBI differ in white matter microstructure compared to the two control groups. Due to the lack of imaging literature in chronic pediatric mTBI, we expected lower FA and higher mean diffusivity (MD) in the investigated tracts, as is often the case for the chronic phase of adult mTBI (Lindsey et al., 2021).

## Methods

### Study design

The ABCD study is a prospective, longitudinal study funded by the National Institute of Health (NIH) and has been conducted at 21 sites in the United States. Children at the age of 9–10 years were recruited from schools with demographically diverse backgrounds and, with the study still ongoing, will be followed for 10 years with yearly follow-ups including various demographic, cognitive, behavioral, and neuroimaging assessments (Garavan et al., 2018; Volkow et al., 2018). Written consent was obtained from the parents. The total available sample size at the time of this analysis was 11 876 for the baseline assessment, 10 414 for the 2-year, and 6251 for the 3-year follow-up. For this study, ABCD Data release 4.0 is used for all data except for diffusion MRI, where data from release 3.0 were harmonized before release 4.0 was available. Raw data are available for researchers upon request at https://nda.nih.gov/abcd/.

### Study sample

#### Mild traumatic brain injury

Parents completed the ABCD Parent Ohio State Traumatic Brain Injury Screen-Short Modified (OTBI; Corrigan & Bogner, 2007), which asks about a series of events relevant to TBI (e.g. ‘Has your child ever been hospitalized or treated in an emergency room following an injury to his/her head or neck?’ or ‘Has your child ever injured his/her head or neck in [one of several injury mechanisms]?’). If the item is endorsed, parents report if and for how long the child suffered from (1) loss of consciousness (LOC) or (2) amnesia and/or an altered mental state. Children were categorized as having sustained a ‘possible mTBI’ (amnesia/altered mental state without LOC) or ‘mTBI’ (with LOC) by the ABCD study team. For this study, both groups were included in the mTBI group based on current criteria for the clinical diagnosis of mTBI in children and adolescents in which LOC is not required (Kay et al., 1993). Parents then provided the age (in years) at which the event occurred. Children with a moderate or severe TBI before (*n* = 2) and after baseline (*n* = 1) were excluded.

#### Control group selection

Children with a history of mTBI were compared to both TDC and OI controls. Participants in the mTBI group were matched to the two different control groups for age, sex, family income, race, and study site using the ‘optimal matching’ from the MatchIt R-package (Ho, Imai, King, & Stuart, 2011). A ratio of 1:2 (1 mTBI case matched to 2 control cases for each control group) was chosen for each control group because only ~1200 children were eligible for the OI group and differing control group sizes would have limited comparability.

#### Missing data

Some children had missing data on covariates of interest (e.g., parents chose not to report their income), reducing the mTBI sample size to *n* = 406 (*n*_TDC_ = 812, *n*_OI_ = 812). Additionally, cognitive and behavioral outcome measures were assessed at follow-up, so that children would have sustained their first mTBI at least 2 years before. Because data were not available for all children at this point, the sample size of mTBI and control groups varies between outcome variables. Each respective sample size can be found in Table 1 and is depicted as a flowchart in online Supplementary Fig. S1.

**Table 1.** Overview of dependent variables

Measure	Domain	Type of measurement	Time-point	*n*_mTBI_ (*n*_TDC_, *n*_OI_)
NIH Flanker	Executive function	Neuropsychological test	2-year FU	271 (547, 569)
BDEFS	Executive function	Parent-report	3-year FU	224 (444, 427)
UPPS-P	Impulsivity	Self-report	2-year FU	362 (715, 713)
BIS/BAS *Fun Seeking*	Impulsivity	Self-report (subscale)	2-year FU	362 (715, 713)
DERS-P 1	Emotion regulation	Parent-report (subscale)	3-year FU	220 (430, 424)
DERS-P 4	Emotion regulation	Parent-report (subscale)	3-year FU	220 (430, 424)
Diffusion (FA and MD): CB SLF II/III CC 1-7	White matter microstructure	Diffusion tensor imaging (dMRI)	Baseline	321 (636, 656)

*Note*. An overview of dependent variables for the examined domains with the respective type of measurement, the time-point of assessment and the available sample size. mTBI, mild traumatic brain injury; TDC, typically developing children; OI, orthopedic injury; FU, follow-up; BDEFS, *Barkley Deficits in Executive Functioning Scale*; NIH Flanker, *NIH Toolbox Flanker Inhibitory Control and Attention Test*; UPPS-P, *UPPS-P Impulsive Behavior Scale*; BIS/BAS, *Behavioral Inhibition/Behavioral Approach System*; DERS-P, *Difficulties in Emotion Regulation Scale*; FA, fractional anisotropy; MD, mean diffusivity; CB, cingulum bundle (left/right included); SLF, superior longitudinal fasciculus (left/right included); CC, corpus callosum (connecting hemispheres).

### Measures

#### Demographic variables

Demographic measures include biological sex (female/male), age at baseline (in months), race (American Indian and Alaska Native/Asian/Black/Multiple/Native Hawaiian and Other Pacific Islander/Other/White), and total family income. Family income in the past year was reported on an ordinal scale from 1 (‘less than $5000’) to 10 (‘$200 000 and greater’) and used as a proxy for socio-economic status (SES). Body mass index (BMI) was calculated. Additionally, handedness was assessed using the Youth Edinburgh Handedness Inventory Short Form (EHIS; Oldfield, 1971).

#### Cognitive and behavioral variables

*Executive functioning*. For executive functioning, two assessment modalities were used: The first assessment was the *Barkley Deficits in Executive Functioning Scale* (BDEFS; Barkley, 2012), which is a parent-rating scale of executive functioning in the activities of daily life. The short form used for the ABCD study is comprised of 14 items that are scored from 1 (‘never or rarely’) to 4 (‘very often’) and scores were summed for the analysis. The second assessment for executive functioning was the *NIH Toolbox Flanker Inhibitory Control and Attention Test* (NIH Flanker), which is a neuropsychological test of attention and inhibition (Gershon et al., 2013). Here, children focus on one stimulus while inhibiting the shifting of attention to other stimuli flanking the target. For the NIH toolbox, standard scores are available and therefore, the age-corrected score was used. Regarding the other cognitive/behavioral outcomes, no normed scores were provided by the ABCD study and raw scores were used instead.

*Impulsivity*. Two self-report measures were used to assess impulsivity. The *Abbreviated Youth Version of the UPPS-P* (*urgency, premeditation, perseverance, sensation seeking, and positive urgency*) *Impulsive Behavior Scale* (Watts, Smith, Barch, & Sher, 2019) consists of 20 items rated from 1 (‘not at all like me’) to 4 (‘very much like me’). The sum of all items was used in this analysis. Additionally, the *Fun Seeking* subscale from the *Youth Behavioral Inhibition/Behavioral Approach System* (BIS/BAS; Carver & White, 1994) was used as it provides a more nuanced assessment. This subscale of the BIS/BAS contains four items scored from 0 (‘not true’) to 3 (‘very true’) and is specifically related to reward reactivity and impulsivity (Smillie, Jackson, & Dalgleish, 2006).

*Emotion regulation*. Emotion regulation was assessed using the parent-report version of the *Difficulties in Emotion Regulation Scale* (DERS-P; Bunford et al., 2020). Only two of the four subscales (factors) target elements of control and self-regulation and were used in the current analysis: Factor 1 reflects feelings of losing control when faced with negative emotions (*Catastrophize*, 11 items), and Factor 4 reflects not being able to focus one's thoughts when experiencing strong emotions (*Distracted*, four items). The other two factors target the experience of negative secondary emotions (*Negative secondary*) and the recognition of one's own emotions (*Attuned*) and were not included in this study. Items were scored from 0 (‘almost never’) to 5 (‘almost always’).

#### Diffusion MRI and white matter microstructure

MRI testing in the ABCD study is performed according to a standardized protocol. Details on image acquisition are reported elsewhere (Casey et al., 2018). Data with insufficient quality as assessed by experts were excluded (Hagler et al., 2019). Because diffusion MRI is particularly sensitive to non-linear site- and scanner-specific effects (Mirzaalian et al., 2016), additional harmonization was performed across different scanners before the data were pooled and analyzed statistically. To this end, the minimally processed dMRI data (3.0 release) were harmonized with our well-validated approach based on rotation invariant spherical harmonics (RISH, https://github.com/pnlbwh/dMRIharmonization; Cetin-Karayumak et al., 2019, 2020). Details on the specifics of the harmonization used in the current study are reported by Cetin-Karayumak et al. (2023b). We note that diffusion data acquired on MRI scanners manufactured by Philips did not pass quality control criteria during harmonization (Cetin-Karayumak et al., 2023b), which led to the exclusion of *n* = 38 children with mTBI, *n* = 75 TDC and *n* = 80 OI controls from imaging analyses. The final sample size for dMRI analyses used in this study was *n* = 321 children with mTBI (*n* = 636 TDC, *n* = 656 OI).

After obtaining the harmonized dMRI data, whole brain tractography was performed using an advanced, multi-tensor unscented Kalman filter (UKF, https://github.com/pnlbwh/ukftractography; Malcolm, Shenton, & Rathi, 2010) for each subject under study. Tractography data were then automatically segmented into 73 white matter tracts using an anatomically curated atlas (https://github.com/SlicerDMRI/whitematteranalysis; Zhang et al., 2018). For this analysis, 13 tracts were selected based on their reported association with mTBI, executive functioning, and impulsivity (Cardenas-Iniguez et al., 2022; Lindsey et al., 2021; Owens et al., 2020), including the left and right cingulum bundle (CB), two posterior sections of the superior longitudinal fasciculus (left and right SLF, II and III), and the corpus callosum divided into seven sections (CC 1–7). FA (directionality of diffusion) and MD (mean diffusivity in all directions) are the most used measures of white matter pathology associated with mTBI (Shenton et al., 2012) and were analyzed for each of the aforementioned tracts.

### Statistical analyses

For comparability between measures, cognitive/behavioral variables and white matter microstructure were *z*-standardized before further analysis.

#### Group differences

All analyses were performed using R version 4.1.2 (R Core Team, 2021); required packages are listed in the publicly available R-script (https://github.com/anjabetz/ABCD–mTBI). Linear regression models were performed for each of the outcomes with group (mTBI as the reference, TDC and OI) as the independent variable and with age at baseline, sex, race, SES, and site as covariates. To test for group differences in FA and MD, BMI and handedness were included as additional covariates. *p*-Values were corrected for multiple comparisons using a false-discovery-rate of 0.05 (FDR; Benjamini & Hochberg, 1995) separately for cognitive/behavioral outcomes (12 comparisons), FA (26 comparisons), and MD (26 comparisons). *F^2^* was calculated as an effect size (Cohen, 1988).

#### Age-at-injury

To examine whether group differences between mTBI and TDC were dependent on the age at which the mTBI was sustained, the mTBI group was divided into three groups based on their retrospective age at the time they sustained the first mTBI (age-at-time of injury in years grouped in: ‘0–3’, ‘4–7’, and ‘8–10’). The matched pairs were extracted and each age-at-injury group was compared to their respective matches from the TDC group. This kept the sample ratio at 1:2 (mTBI:TDC) and allowed us to investigate non-linear effects with a simple linear model. The age groups chosen were small enough to allow for investigation of sensitive periods but large enough to be interpreted in a developmental context. The OI control group was not included in this part of the analysis, because children in this group *had* sustained an injury at a certain age, but that age was not assessed in the ABCD study and could neither be controlled for nor grouped together.

## Results

### Demographics

A total of 448 children sustained an mTBI before the ABCD baseline assessment, of which 406 had complete data on covariates (i.e. age, sex, race, SES, and site). This cohort of mTBI subjects was used for the analyses and matched to TDC and OI controls (812 children in each control group). Demographic characteristics are summarized in Table 2. The groups did not differ in any of the demographical variables due to the matching procedure; a comparison of mTBI to the total ABCD baseline sample has already been reported (Dufour, Adams, Brody, Puente, & Gray, 2020).

**Table 2.** Demographic characteristics

	mTBI (*n* = 406)	TDC (*n* = 812)	OI (*n* = 812)	*p*-value (TDC/OI)
Age (mean in months [s.d.])	119.98 (7.53)	120 (7.62)	119.74 (7.62)	0.955/0.598
Biological sex [% male/female]	61.08/38.92	60.96/39.04	58.00/42.00	1/0.333
Handedness [%]	80/7/13	80/8/13	80/6/14	0.816/0.736
BMI (mean [s.d.] kg/m^2^)	18.52 (4.02)	18.73 (4.09)	18.72 (4.37)	0.399/0.435
Race (*n* [%])				0.993/0.910
White	297 (73.15)	593 (73.03)	599 (73.77)	
Black	37 (9.11)	72 (8.87)	74 (9.11)	
Asian	6 (1.48)	14 (1.72)	16 (1.97)	
NHPI	0 (0.00)	0 (0.00)	0 (0.00)	
AIAN	0 (0.00)	0 (0.00)	0 (0.00)	
Other	19 (4.68)	42 (5.17)	30 (3.69)	
Multiple	47 (11.58)	91 (11.21)	93 (11.45)	
Total family income (*n* [%])				0.972/0.983
Less than $5000	9 (2.22)	23 (2.83)	20 (2.46)	
$5000 through $11 999	10 (2.46)	21 (2.59)	18 (2.22)	
$12 000 through $15 999	6 (1.48)	8 (0.99)	10 (1.23)	
$16 000 through $24 999	18 (4.43)	29 (3.57)	30 (3.69)	
$25 000 through $34 999	19 (4.68)	38 (4.68)	39 (4.80)	
$35 000 through $49 999	25 (6.16)	49 (6.03)	46 (5.67)	
$50 000 through $74 999	61 (15.02)	114 (14.04)	115 (14.12)	
$75 000 through $99 999	55 (13.55)	130 (16.01)	127 (15.64)	
$100 000 through $199 999	142 (34.97)	280 (34.48)	298 (36.70)	
$200 000 and greater	61 (15.02)	120 (14.78)	109 (13.42)	
Months since injury (mean [s.d.])	46.31 (32.15)	–	–	
Age at first mTBI (n [%])				
0–3 years	91 (22.52)	–	–	
4–7 years	147 (36.38)	–	–	
8–10 years	166 (41.09)	–	–	

*Note*. mTBI, mild traumatic brain injury; TDC, typically developing children; OI, orthopedic injury; NHPI, Native Hawaiian and other Pacific Islander; AIAN, American Indian and Alaska Native. Handedness % in right-/left-handed/mixed. Percentages were rounded and therefore may not add up to 100.

### Group differences in cognition and behavior

**Figure 1.**
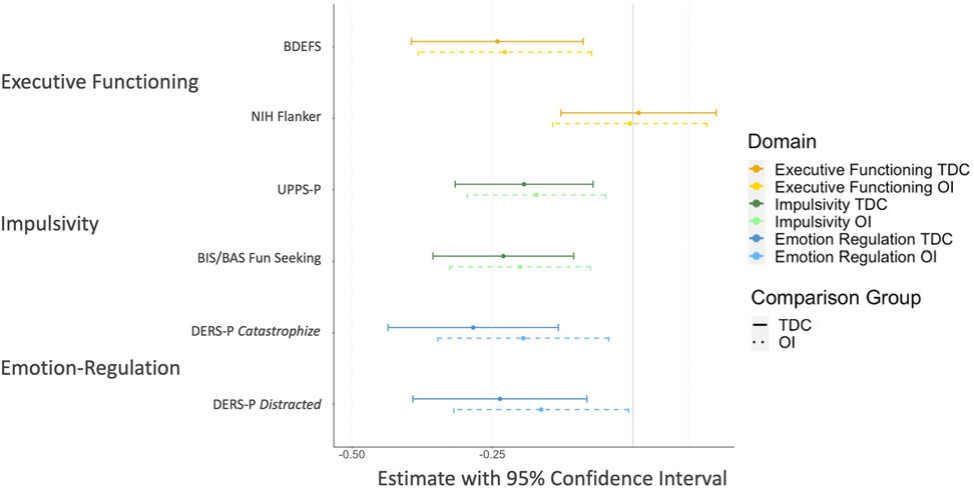
Differences in cognition and behavior. Estimate of the standardized *β*-coefficients for all cognitive and behavioral variables. These represent the estimated change from the mTBI group (reference, therefore here represented by the vertical 0-line) to the respective control group. Horizontal bars reflect the estimate's 95% confidence interval. Variables with confidence intervals not including 0 are considered significant (FDR-corrected *p* < 0.05).

Standardized regression coefficients with 95% confidence intervals are depicted in Fig. 1; coefficients, test statistics, degrees of freedom, corrected *p*-values, and effect sizes can be found in Table 3. The control groups did not differ on any outcome variables.

**Table 3.** Inferential statistics for cognition and behavior

Variable	df	Test statistic	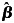 [95% CI]	*p*-value	*f*^2^
BDEFS					
mTBI *v*. TDC	1057	-3.09	-0.24 [-0.39, -0.09]	0.005*	0.009
mTBI *v*. OI	1057	-2.90	-0.23 [-0.38, -0.07]	0.007*	0.008
NIH Flanker					
mTBI *v*.TDC	1349	0.14	0.01 [-0.13, 0.15]	0.937	<0.000
mTBI *v*. OI	1349	-0.08	-0.01 [-0.14, 0.13]	0.937	<0.000
UPPS-P					
mTBI *v*.TDC	1752	-3.10	-0.19 [-0.32, -0.07]	0.005*	0.005
mTBI *v*. OI	1752	-2.75	-0.17 [-0.29, -0.05]	0.009*	0.004
BIS/BAS *Fun Seeking*					
mTBI *v*. TDC	1752	-3.61	-0.23 [-0.36, -0.11]	0.002*	0.007
mTBI *v*. OI	1752	-3.14	-0.20 [-0.33, -0.08]	0.005*	0.006
DERS-P *Catastrophize*					
mTBI *v*. TDC	1036	-3.68	-0.28 [-0.44, -0.13]	0.002*	0.013
mTBI *v*. OI	1036	-2.52	-0.20 [-0.35, -0.04]	0.016*	0.006
DERS-P *Distracted*					
mTBI *v*. TDC	1036	-3.00	-0.24 [-0.39, -0.08]	0.006*	0.009
mTBI *v*. OI	1036	-2.06	-0.16 [-0.32, -0.01]	0.048*	0.004

*Note*. Sample size of mTBI and respective control groups varies due to data availability at follow-up. mTBI is used as the reference for regression coefficients. df, Residual degrees of freedom; TDC, typically developing controls; OI, orthopedic injury; BDEFS, *Barkley Deficits in Executive Functioning Scale*; NIH Flanker, *NIH Toolbox Flanker Inhibitory Control and Attention Test*; UPPS-P, *UPPS-P Impulsive Behavior Scale*; BIS/BAS, *Behavioral Inhibition/Behavioral Approach System Fun Seeking Scale*; DERS-P, *Difficulties in Emotion Regulation Scale.* *Indicates significant *p*-values at *p* < 0.05 after correction.

#### Executive functioning

Children with a history of mTBI displayed significantly higher parent-rated executive *dysfunction* on the BDEFS than either of the control groups (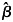
_TDC_ = -0.24, *p*_TDC_ = 0.005
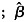
_OI_ = -0.23, *p*_OI_ = 0.007). The NIH Flanker task, as a neuropsychological test of executive functioning, did not differ among the groups (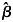
_TDC_ = 0.01, *p*_TDC_ = 0.937; 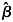
_OI_ = -0.01, *p*_OI_ = 0.937).

#### Impulsivity

Impulsivity as assessed with the UPPS-P was significantly higher in the mTBI group compared to both control groups (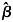
_TDC_ = -0.19, *p*_TDC_ = 0.005; 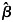
_OI_ = -0.17, *p*_OI_ = 0.009), as was the score on the BIS/BAS *Fun Seeking* Scale (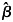
_TDC_ = -0.23, *p*_TDC_ = 0.002; 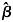
_OI_ = -0.20, *p*_OI_ = 0.005).

#### Emotion regulation

Regarding emotional self-regulation, the first DERS-P factor (*Catastrophize*) was significantly higher in the mTBI group compared to both control groups (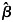
_TDC_ = -0.28, *p*_TDC_ = 0.002; 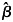
_OI_ = -0.20, *p*_OI_ = 0.016), as was the fourth factor (*Distracted*) (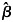
_TDC_ = -0.24, *p*_TDC_ = 0.006; 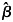
_OI_ = -0.16, *p*_OI_ = 0.048).

### Group differences in white matter microstructure

Analyses of white matter microstructure did not reveal any significant differences in FA or MD between the groups in the CB, the SLF, or the CC (all *p*-values > 0.05, see online Supplementary Tables S2 and S3).

### Influence of age-at-injury

For this analysis, children with a history of mTBI were split into three age-at-injury categories: 0–3 years (*n* = 91), 4–7 years (*n* = 147), and 8–10 years (*n* = 166). These groups were compared to their respective matched TDC controls. At baseline, children had a mean time-since-injury of 46.31 (s.d. = 32.15) months.

**Figure 2.**
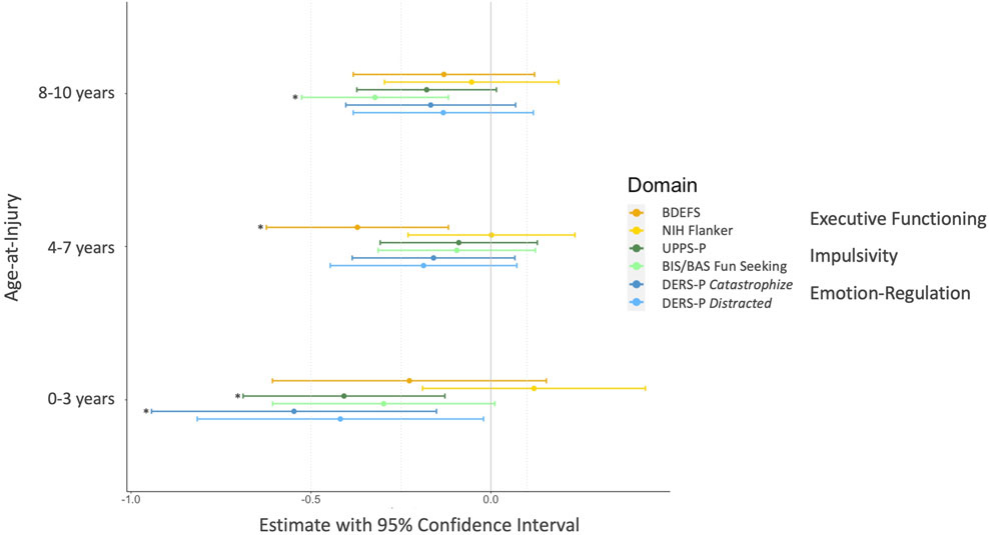
Effect of age-at-injury on cognition and behavior. Comparison of three separate groups based on age-at-injury (0–3, 4–7, 8–10 years, each represented by the vertical 0 line) to their respective matched TDC control group. Estimate of the standardized *β*-coefficients for all cognitive and behavioral variables. *Indicate FDR-corrected *p*-values < 0.05.

The difference on the BDEFS was mainly driven by the group with an mTBI between the ages of 4-7 years (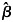 = 0.37, *p* = 0.029). The difference on the BIS/BAS *Fun Seeking* Scale was driven by the group with their first mTBI between 8-10 years (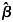 = 0.32, *p* = 0.029), while the difference on the UPPS-P was driven by the age group 0-3 (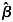 = 0.41, *p* = 0.029). Differences in the DERS Factor 1 (*Catastrophize*) were driven by the age group 0-3 (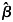 = 0.55, *p* = 0.035). No other age groups differed from controls on these measures. No significant differences depending on the age-at-injury were observed on the NIH Flanker Task and on the DERS Factor 4 (see online Supplementary Table S4). Effects of age-at-injury on cognitive and behavioral variables are depicted in Fig. 2.

Regarding white matter, no differences from controls emerged when examining different ages-at-injury (see online Supplementary Tables S5 and S6).

## Discussion

Children with a history of mTBI showed worse executive function and emotion regulation as well as higher impulsivity compared to controls. In addition, the age of the child at the time of injury influenced which domain was affected by mTBI. That is, children showed more emotional dysregulation if they sustained an mTBI between the age of 0 and 3 years, more executive dysfunction if they sustained an mTBI between the age of 4 and 7 years, and more impulsivity than TDC if they sustained an mTBI between the age of 0 and 3 or 8 and 10 years. This study found no differences between groups in task-based executive functioning. Finally, there were no group differences in diffusion measures of the investigated white matter tracts (i.e., CB, CC, and SLF).

### Executive function, impulsivity, and emotion-regulation

Children with a history of mTBI displayed more executive dysfunction, difficulties in emotion regulation, and impulsivity based on parent- and self-report compared to TDC and OI controls. In line with our results, Jones et al. (2021) report difficulties in executive, emotional, and behavioral domains 7 years following an mTBI. Another study reports psycho-emotional problems that persist up to 2 years following a TBI (Ewing-Cobbs et al., 2021). However, still other studies report behavioral difficulties that had either subsided 1 year after mTBI (Maillard-Wermelinger et al., 2009) or parent-rated executive functioning that showed an improving trajectory 2 years following TBI (Keenan, Clark, Holubkov, Cox, & Ewing-Cobbs, 2021). One possible explanation for these inconsistent findings in the literature may be that there is a non-linear course of recovery. That is, after an initial alleviation of symptoms, new problems may arise with new developmental milestones and increasing demands from the child's environment. Future studies using longitudinal study designs with multiple follow-up assessments over a longer period are needed to fully address the effect of mTBI on executive function, emotion regulation, and impulsivity, i.e. studies should not focus on only individuals in the acute phase of post-injury but also over a period of time as new developmental challenges emerge.

While there is executive dysfunction in children with a history of mTBI based on parent report, this study found no group differences in executive functioning as assessed by neuropsychological testing. This is in line with a previous report on the same dataset (Dufour et al., 2020), which also did not find associations between mTBI and principal cognitive components derived from the NIH toolbox tests. Of note, neuropsychological test performance has previously been shown to account for less than 20% of the variance in parent-ratings after adolescent mTBI (Lace et al., 2019). This does, however, not necessarily mean that they differ regarding quality of assessment. Rather, they appear to be tapping into different aspects with neuropsychological tests reflecting the ‘cold’ (i.e., cognitive, controlled, and under laboratory conditions) side of executive functioning while ratings provided by parents may also include ‘hot’ elements (e.g., emotional distress, risky behavior in everyday life) (Lace et al., 2019). In the context of this study, this may explain why difficulties in emotion-regulation were found in addition to higher parent-rated executive dysfunction; both would include day-to-day abilities of self-regulation. To capture the wide concept of executive functioning, studies should ideally include multi-modal testing.

Of note, tests often isolate one cognitive ability while ratings can capture a broader scope of everyday function. This study used a neuropsychological test of inhibition, because inhibition as opposed to other forms of executive functioning has shown a sensitivity for age-at-injury effects (Resch et al., 2019). However, problems with inhibition have previously been shown to subside even while other deficits persist (Keenan et al., 2021). It is therefore possible that other domains of executive functioning would have captured differences when using task-based measures.

Of further note, while performance on neuropsychological testing did not differ between mTBI and control groups, the reported behavioral alterations may indirectly affect a child's academic functioning. That is, more impulsive and unregulated behavior could lead to difficulties in coordinating homework and studying, which children need to do more independently as they develop. Academic achievement in childhood and adolescence is also associated with self-reported executive functioning (van Tetering, Jolles, van der Elst, & Jolles, 2022) and teacher-reported self-regulation (van Tetering, de Groot, & Jolles, 2018). Further investigations using external criteria such as grades in addition to parent-report and neuropsychological testing are therefore warranted.

### Effect of age-at-injury

In pediatric TBI, there are differing perspectives on the influence of age-at-injury. Assuming a linear relationship, a younger age is often considered a risk factor for worse outcomes following a moderate-severe TBI (Goh et al., 2021). However, after a mild TBI, such clear associations have not been demonstrated (Anderson, Catroppa, Morse, Haritou, & Rosenfeld, 2005). An alternative view is the model of sensitive developmental periods (Anderson et al., 2009; Zamani, Ryan, Wright, Caeyenberghs, & Semple, 2020), suggesting that age-at-injury does not show a ‘one size fits all’ relationship for cognitive, behavioral, and structural domains. Rather, an mTBI likely impacts children differentially based on developmental stage. Functions that are rapidly developing at time of injury are at increased risk for long-term impairments (Zamani et al., 2020). Interestingly, in this study, higher emotional dysregulation was present in children who sustained an mTBI between the ages of 0 and 3. This is in line with a review article on self-regulation and effortful control, which points out that the first few years of life are most relevant for children's emotional self-regulation. Afterwards, interindividual differences remain more stable (Eisenberg, Spinrad, & Eggum, 2010). With regard to executive functioning, most studies show that associated cognitive abilities develop most rapidly during the ages of 5 to 8 (Goldstein & Naglieri, 2014). While there is less research on behavioral executive functioning, the results of this study match this age range by showing that parent-rated executive functioning is mainly affected by children who experience an mTBI between the ages of 4 and 7. Impulsivity, on the other hand, was affected if the mTBI was sustained both between the ages of 0 and 3 or 8 and 10. In the literature, the development of impulsivity cannot be pinpointed as clearly as for other functions. A head injury before the age of 5 has previously been shown to attenuate developmental declines in impulsivity (Fullerton, Jackson, Tuvblad, Raine, & Baker, 2019). On the other hand, studies showed that the later elementary school years (i.e., 8-11 years) are a transitional period for the development of impulse control (Chen et al., 2021). A more complex explanation may be that the UPPS reflects general impulsivity, which starts to decline already in early childhood (Schwartz, Connolly, & Alsolami, 2022) and therefore was implicated by mTBI before 3 years. The BIS/BAS Fun Seeking scale would reflect sensation seeking specifically, which still appears to increase in late childhood (Schwartz et al., 2022) and could be affected by the mTBI between 8 and 10 years. Taken together, our results emphasize the importance of taking age-at-injury into account as a complex, presumably non-linear influence.

### White matter microstructure

To investigate the neurophysiological mechanisms underlying the behavioral differences reported above, we examined white matter microstructure in several tracts that have previously been associated with mTBI, executive functioning, and the related behavior. To account for non-linear scanner effects, we used the newly available diffusion MRI data that were harmonized across study sites (Cetin-Karayumak et al., 2023b). The effectiveness of this harmonization approach has been demonstrated in several neuroimaging studies (e.g., Cetin Karayumak, Kubicki, & Rathi, 2018, Cetin-Karayumak et al., 2020, 2023a; De Luca et al., 2022; Di Biase et al., 2021; Elad et al., 2021; Lv et al., 2021; Seitz et al., 2021; Ye et al., 2021).

There were no differences detected between children with mTBI and the two control groups in FA and MD. Previous studies have reported mixed results regarding the effect of pediatric mTBI on white matter microstructure (Jain et al., 2021). It is possible that mild injuries like the ones included in this study do not lead to changes in white matter microstructure (Ware et al., 2020). Alternatively, any initial changes may already have subsided by the time of scanning (Van Beek, Vanderauwera, Ghesquière, Lagae, & De Smedt, 2015) with children having sustained their first mTBI an average of 4 years before baseline. Further, if changes in white matter microstructure are highly individual, they also may not be captured using group comparisons. In future studies, comparing individual cases to an atlas of normative data may be more sensitive for detecting white matter alterations at the level of the individual (Bouix et al., 2013).

### Limitations

There are several limitations to the study that need to be considered. First, this analysis is cross-sectional. The trajectory of the reported difficulties could not be assessed, so it remains to be clarified whether they begin shortly after an injury and persist or whether they occur only with progressing development. Moreover, pre-injury characteristics were not available and thus could not be controlled for. It is possible that group differences were at least partly present before children sustained an mTBI. We addressed this by matching the groups carefully for age, sex, race, income, and study site, statistically controlling for these factors, and including an OI control group. This should reduce the influence of demographic, pre-injury, and general injury characteristics, but cannot completely exclude them. The analysis would have profited from an OI group that was also divided according to age-at-injury of the mTBI group. Unfortunately, this information was not provided by the ABCD medical history questionnaire. In this study, information on mTBI was based on parent-report. Therefore, the data may be subject to recall bias regarding injuries at all, their mechanism, the subsequent symptoms, or the child's age. However, this approach also offers the strength of capturing injuries that may have been on the milder spectrum and thus, not presented to a physician at time of injury. The corresponding effect sizes were very small, which is in part expected in such large population studies (Owens et al., 2021). Given the mild injuries, the time since injury and the fact that based on the literature, only a subset of participants may experience lasting symptoms (Babcock et al., 2013), we still consider them clinically meaningful and important to report. Finally, children from certain racial and lower socio-economic backgrounds were under-represented in the current sample and had disproportionately high missing outcome values. While this was not to be avoided, it may limit generalizability and also prohibited the investigation of more complex interaction effects.

## Conclusion

Children with a history of mTBI showed more executive dysfunction, more impulsivity, and more difficulties in emotional self-regulation compared to TDC and OI controls. Moreover, age-at-injury has an effect suggesting that there may be sensitive periods in brain development. White matter microstructure did not differ between groups. To address this, future studies should consider investigating white matter alterations at the level of the individual.

## Supporting information

Betz et al. supplementary material Betz et al. supplementary material

## Supplementary material

The supplementary material for this article can be found at https://doi.org/10.1017/S0033291724000229
