# Supplementary material for: Executive functioning, behavior, and white matter microstructure in the chronic phase after pediatric mild traumatic brain injury: results from the adolescent brain cognitive development study
Source: Psychol Med. 2024 Mar 18;54(9):2133–43. doi: 10.1017/S0033291724000229 (PMC11413348; doi:10.1017/S0033291724000229)
Supplement: Betz et al. supplementary material [file S0033291724000229sup001.docx]

**Executive Functioning, Behavior, and White Matter Microstructure
in the Chronic Phase after Pediatric Mild Traumatic Brain Injury:
Results from the Adolescent Brain Cognitive Development Study**

Online-only Supplement

**Supplementary Figure S1**

Flowchart of the available sample size for each part of the analysis
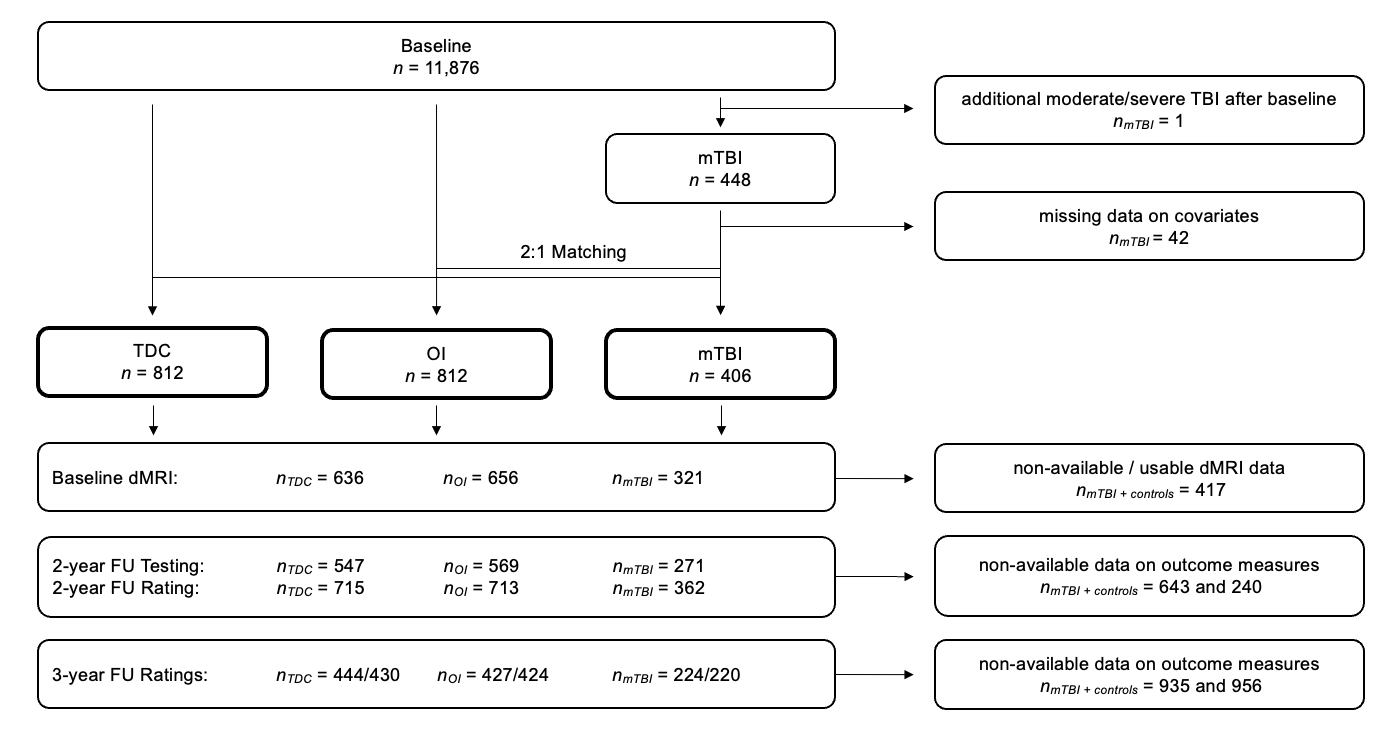


**Supplemental Table S2**

*Inferential Statistics for Group Differences in Fractional Anisotropy*

| Tract |  | Df | Test Statistic | $\hat{\beta}$ | 95% CI | *p* | *f^2^* |
| --- | --- | --- | --- | --- | --- | --- | --- |
| CB left | | | | | | | |
|  | TDC | 1575 | -0.54 | 0.0268 | [-0.17;0.09] | .972 | 0.0002 |
|  | OI | 1575 | 0.41 | -0.0989 | [-0.10;0.16] | .972 | 0.0001 |
| CB right | | | | | | | |
|  | TDC | 1575 | -1.52 | -0.0216 | [-0.23;0.03] | .972 | 0.0015 |
|  | OI | 1575 | -0.33 | 0.0287 | [-0.15;0.11] | .972 | 0.0001 |
| SLF II left | | | | | | | |
|  | TDC | 1556 | 0.44 | 0.0540 | [-0.10;0.16] | .972 | 0.0001 |
|  | OI | 1556 | 0.82 | -0.0177 | [-0.08;0.18] | .972 | 0.0004 |
| SLF II right | | | | | | | |
|  | TDC | 1556 | -0.26 | 0.0796 | [-0.15;0.12] | .972 | <0.0001 |
|  | OI | 1556 | 1.18 | -0.0081 | [-0.05;0.21] | .972 | 0.0009 |
| SLF III left | | | | | | | |
|  | TDC | 1556 | -0.13 | 0.0078 | [-0.14;0.12] | .972 | <0.0001 |
|  | OI | 1556 | 0.12 | 0.0641 | [-0.12;0.13] | .972 | <0.0001 |
| SLF III right | | | | | | | |
|  | TDC | 1556 | 0.96 | 0.0778 | [-0.07;0.20] | .972 | 0.0006 |
|  | OI | 1556 | 1.17 | -0.0214 | [-0.05;0.21] | .972 | 0.0009 |
| CC 1 | | | | | | | |
|  | TDC | 1556 | -0.32 | 0.0354 | [-0.15;0.11] | .972 | 0.0001 |
|  | OI | 1556 | 0.53 | -0.0349 | [-0.10;0.17] | .972 | 0.0002 |
| CC 2 | | | | | | | |
|  | TDC | 1575 | -0.55 | 0.0022 | [-0.16;0.09] | .972 | 0.0002 |
|  | OI | 1575 | 0.04 | -0.0800 | [-0.12;0.13] | .972 | <0.0001 |
| CC 3 | | | | | | | |
|  | TDC | 1556 | -1.2 | 0.0066 | [-0.21;0.05] | .972 | 0.0009 |
|  | OI | 1556 | 0.1 | -0.0215 | [-0.12;0.14] | .972 | <0.0001 |
| CC 4 | | | | | | | |
|  | TDC | 1575 | -0.34 | 0.0027 | [-0.15;0.10] | .972 | 0.0001 |
|  | OI | 1575 | 0.04 | 0.0501 | [-0.12;0.13] | .972 | <0.0001 |
| CC 5 | | | | | | | |
|  | TDC | 1556 | 0.78 | 0.0272 | [-0.08;0.18] | .972 | 0.0004 |
|  | OI | 1556 | 0.42 | 0.1082 | [-0.10;0.15] | .972 | 0.0001 |
| CC 6 | | | | | | | |
|  | TDC | 1556 | 1.7 | 0.0501 | [-0.02;0.23] | .972 | 0.0018 |
|  | OI | 1556 | 0.79 | 0.0653 | [-0.08;0.18] | .972 | 0.0004 |
| CC 7 | | | | | | | |
|  | TDC | 1556 | 1 | 0.0286 | [-0.06;0.19] | .972 | 0.0006 |
|  | OI | 1556 | 0.44 | -0.0355 | [-0.10;0.16] | .972 | 0.0001 |

*Note.* Inferential statistics for group differences in fractional anisotropy. Abbreviations: Df = Residual degrees of freedom, CI = confidence interval, CB = cingulum bundle, SLF = superior longitudinal fasciculus, CC = corpus callosum.

**Supplementary Table S3**

*Inferential Statistics for Group Differences in Mean Diffusivity*

| Tract |  | Df | Test Statistic | $\hat{\beta}$ | 95% CI | *p* | *f^2^* |
| --- | --- | --- | --- | --- | --- | --- | --- |
| CB left | | | | | | | |
|  | TDC | 1575 | -0.83 | -0.0544 | [-0.18;0.07] | .759 | 0.0004 |
|  | OI | 1575 | -0.97 | -0.0634 | [-0.19;0.07] | .759 | 0.0006 |
| CB right | | | | | | | |
|  | TDC | 1575 | -1.11 | -0.0729 | [-0.20;0.06] | .759 | 0.0008 |
|  | OI | 1575 | -0.30 | -0.0193 | [-0.15;0.11] | .903 | 0.0001 |
| SLF II left | | | | | | | |
|  | TDC | 1556 | 1.26 | 0.0883 | [-0.05;0.23] | .759 | 0.0010 |
|  | OI | 1556 | 1.25 | 0.0878 | [-0.05;0.23] | .759 | 0.0010 |
| SLF II right | | | | | | | |
|  | TDC | 1556 | -0.30 | -0.0199 | [-0.15;0.11] | .903 | 0.0001 |
|  | OI | 1556 | 1.13 | 0.0746 | [-0.06;0.21] | .759 | 0.0008 |
| SLF III left | | | | | | | |
|  | TDC | 1556 | -0.77 | -0.0514 | [-0.18;0.08] | .759 | 0.0004 |
|  | OI | 1556 | 0.26 | 0.0169 | [-0.11;0.15] | .903 | <0.0001 |
| SLF III right | | | | | | | |
|  | TDC | 1556 | -0.43 | -0.0279 | [-0.16;0.10] | .903 | 0.0001 |
|  | OI | 1556 | 0.85 | 0.0554 | [-0.07;0.18] | .759 | 0.0005 |
| CC 1 | | | | | | | |
|  | TDC | 1556 | -0.68 | -0.0475 | [-0.18;0.09] | .759 | 0.0003 |
|  | OI | 1556 | -1.48 | -0.1025 | [-0.24;0.03] | .759 | 0.0014 |
| CC 2 | | | | | | | |
|  | TDC | 1575 | -0.15 | -0.0103 | [-0.14;0.12] | .945 | <0.0001 |
|  | OI | 1575 | 0.11 | 0.0077 | [-0.12;0.14] | .945 | <0.0001 |
| CC 3 | | | | | | | |
|  | TDC | 1556 | -0.72 | -0.0492 | [-0.18;0.08] | .759 | 0.0003 |
|  | OI | 1556 | 0.38 | 0.0260 | [-0.11;0.16] | .903 | 0.0001 |
| CC 4 | | | | | | | |
|  | TDC | 1575 | 0.78 | 0.0515 | [-0.08;0.18] | .759 | 0.0004 |
|  | OI | 1575 | 1.28 | 0.0841 | [-0.05;0.21] | .759 | 0.0010 |
| CC 5 | | | | | | | |
|  | TDC | 1556 | 0.29 | 0.0198 | [-0.11;0.15] | .903 | 0.0001 |
|  | OI | 1556 | 0.81 | 0.0545 | [-0.08;0.19] | .759 | 0.0004 |
| CC 6 | | | | | | | |
|  | TDC | 1556 | -1.57 | -0.1066 | [-0.24;0.03] | .759 | 0.0016 |
|  | OI | 1556 | -0.87 | -0.0591 | [-0.19;0.07] | .759 | 0.0005 |
| CC 7 | | | | | | | |
|  | TDC | 1556 | -1.10 | -0.0735 | [-0.21;0.06] | .759 | 0.0008 |
|  | OI | 1556 | 0.02 | 0.0013 | [-0.13;0.13] | .985 | <0.0001 |

*Note.* Inferential statistics for group differences in mean diffusivity. Abbreviations: Df = Residual degrees of freedom, CI = confidence interval, CB = cingulum bundle, SLF = superior longitudinal fasciculus, CC = corpus callosum.

**Supplementary Table S4**

| Variable | Age-at-Injury | Df | Test Statistic | $\hat{\beta}$ | 95% CI | *p* | *f^2^* |
| --- | --- | --- | --- | --- | --- | --- | --- |
| BDEFS |  |  |  |  |  |  |  |
|  | 0-3 | 110 | 1.17 | 0.23 | [-0.16, 0.61] | .402 | 0.0123 |
|  | **4-7** | **219** | **2.87** | **0.37** | **[0.12, 0.63]** | **.029 *** | **0.0377** |
|  | 8-10 | 227 | 1.02 | 0.13 | [-0.12, 0.38] | .430 | 0.0046 |
| NIH Flanker | | | | |  |  |  |
|  | 0-3 | 146 | -0.76 | -0.12 | [-0.43, 0.19] | .507 | 0.0039 |
|  | 4-7 | 266 | -0.01 | -0.00 | [-0.23, 0.23] | .989 | <0.0001 |
|  | 8-10 | 291 | 0.43 | 0.05 | [-0.19, 0.30] | .703 | 0.0006 |
| UPPS-P |  |  |  |  |  |  |  |
|  | **0-3** | **203** | **2.85** | **0.41** | **[0.13, 0.69]** | **.029 *** | **0.0401** |
|  | 4-7 | 360 | 0.80 | 0.09 | [-0.13, 0.31] | .507 | 0.0018 |
|  | 8-10 | 397 | 1.80 | 0.18 | [-0.02, 0.37] | .185 | 0.0082 |
| BIS/BAS Fun Seeking | | | | | |  |  |
|  | 0-3 | 203 | 1.89 | 0.30 | [-0.01, 0.61] | .180 | 0.0176 |
|  | 4-7 | 360 | 0.85 | 0.09 | [-0.12, 0.31] | .507 | 0.0020 |
|  | **8-10** | **397** | **3.09** | **0.32** | **[0.12, 0.53]** | **.029 *** | **0.0241** |
| DERS-P Factor 1 | | |  |  |  |  |  |
|  | **0-3** | **105** | **2.71** | **0.55** | **[0.15, 0.95]** | **.035 *** | **0.0699** |
|  | 4-7 | 212 | 1.38 | 0.16 | [-0.07, 0.39] | .303 | 0.0090 |
|  | 8-10 | 221 | 1.39 | 0.17 | [-0.07, 0.40] | .303 | 0.0087 |
| DERS-P Factor 4 | | | |  |  |  |  |
|  | 0-3 | 105 | 2.06 | 0.42 | [0.02, 0.82] | .150 | 0.0404 |
|  | 4-7 | 212 | 1.41 | 0.19 | [-0.07, 0.45] | .303 | 0.0094 |
|  | 8-10 | 221 | 1.04 | 0.13 | [-0.12, 0.38] | .430 | 0.0049 |

*Inferential Statistics for the Effect of Age-At-Injury on Cognition and Behavior*

*Note.* The respective matched typically developing children group is used as the reference for regression coefficients and compared to mTBI at the ages 0-3, 4-7 and 8-10. * indicate significant *p*-values at *p* < .05 after correction. Abbreviations: Df = Residual degrees of freedom, CI = confidence interval, BDEFS = *Barkley Deficits in Executive Functioning Scale*, NIH Flanker = *NIH Toolbox Flanker Inhibitory Control and Attention Test,* UPPS-P = *UPPS-P Impulsive Behavior Scale*, BIS/BAS = *Behavioral Inhibition/Behavioral Approach System Fun Seeking Scale*, DERS-P = *Difficulties in Emotion Regulation Scale.*

**Supplementary Table S5***Inferential Statistics for the Effect of Age-At-Injury on Fractional Anisotropy*

| Tract | Age-at-Injury | Df | Test statistic | $\hat{\beta}$ | 95% CI | *p* | *f^2^* |
| --- | --- | --- | --- | --- | --- | --- | --- |
| CB left | | | | | | | |
|  | 0-3 | 177 | -0.67 | -0.1107 | [-0.44;0.22] | .908 | 0.0025 |
|  | 4-7 | 309 | 0.79 | 0.0870 | [-0.13;0.30] | .908 | 0.0020 |
|  | 8-10 | 354 | 1.02 | 0.1118 | [-0.11;0.33] | .908 | 0.0029 |
| CB right | | | | | | | |
|  | 0-3 | 177 | -0.79 | -0.1102 | [-0.39;0.17] | .908 | 0.0035 |
|  | 4-7 | 309 | 1.25 | 0.1396 | [-0.08;0.36] | .908 | 0.0051 |
|  | 8-10 | 354 | 1.41 | 0.1599 | [-0.06;0.38] | .908 | 0.0056 |
| SLF II left | | | | | | | |
|  | 0-3 | 176 | -1.18 | -0.1816 | [-0.48;0.12] | .908 | 0.0080 |
|  | 4-7 | 308 | -0.31 | -0.0353 | [-0.26;0.19] | .983 | 0.0003 |
|  | 8-10 | 344 | 0.06 | 0.0068 | [-0.20;0.22] | .990 | <0.0001 |
| SLF II right | | | | | | | |
|  | 0-3 | 176 | -0.96 | -0.1391 | [-0.43;0.15] | .908 | 0.0052 |
|  | 4-7 | 308 | -0.89 | -0.0999 | [-0.32;0.12] | .908 | 0.0026 |
|  | 8-10 | 344 | 1.64 | 0.1943 | [-0.04;0.43] | .908 | 0.0078 |
| SLF III left | | | | | | | |
|  | 0-3 | 176 | -1.15 | -0.1568 | [-0.43;0.11] | .908 | 0.0075 |
|  | 4-7 | 308 | 0.04 | 0.0049 | [-0.22;0.23] | .990 | <0.0001 |
|  | 8-10 | 344 | 1.08 | 0.1204 | [-0.10;0.34] | .908 | 0.0034 |
| SLF III right | | | | | | | |
|  | 0-3 | 176 | -0.44 | -0.0677 | [-0.37;0.24] | .983 | 0.0011 |
|  | 4-7 | 308 | -1.19 | -0.1260 | [-0.36;0.08] | .908 | 0.0046 |
|  | 8-10 | 344 | -0.01 | -0.0014 | [-0.23;0.23] | .990 | <0.0001 |
| CC 1 | | | | | | | |
|  | 0-3 | 176 | -0.01 | -0.0018 | [-0.28;0.28] | .990 | <0.0001 |
|  | 4-7 | 308 | 0.71 | 0.0834 | [-0.15;0.31] | .908 | 0.0017 |
|  | 8-10 | 344 | -0.01 | -0.0015 | [-0.21;0.21] | .990 | <0.0001 |
| CC 2 | | | | | | | |
|  | 0-3 | 177 | 0.15 | 0.0201 | [-0.25;0.29] | .990 | 0.0001 |
|  | 4-7 | 309 | 0.71 | 0.0731 | [-0.13;0.28] | .908 | 0.0016 |
|  | 8-10 | 354 | -0.13 | -0.0147 | [-0.23;0.20] | .990 | 0.0001 |
| CC 3 | | | | | | | |
|  | 0-3 | 176 | 1.18 | 0.1697 | [-0.11;0.45] | .908 | 0.0080 |
|  | 4-7 | 308 | 0.56 | 0.0594 | [-0.15;0.27] | .940 | 0.0010 |
|  | 8-10 | 344 | 0.62 | 0.0725 | [-0.16;0.30] | .908 | 0.0011 |
| CC 4 | | | | | | | |
|  | 0-3 | 177 | 0.66 | 0.0925 | [-0.18;0.37] | .908 | 0.0025 |
|  | 4-7 | 309 | -0.28 | -0.0291 | [-0.24;0.18] | .983 | 0.0002 |
|  | 8-10 | 354 | 0.37 | 0.0420 | [-0.18;0.26] | .983 | 0.0004 |
| CC 5 | | | | | | | |
|  | 0-3 | 176 | 0.75 | 0.1131 | [-0.18;0.41] | .908 | 0.0032 |
|  | 4-7 | 308 | -0.87 | -0.0979 | [-0.32;0.12] | .908 | 0.0024 |
|  | 8-10 | 344 | -0.33 | -0.0357 | [-0.25;0.18] | .983 | 0.0003 |
| CC 6 | | | | | | | |
|  | 0-3 | 176 | -0.77 | -0.1076 | [-0.38;0.17] | .908 | 0.0034 |
|  | 4-7 | 308 | -1.69 | -0.1765 | [-0.38;0.03] | .908 | 0.0092 |
|  | 8-10 | 344 | 0.47 | 0.0526 | [-0.17;0.28] | .983 | 0.0006 |
| CC 7 | | | | | | | |
|  | 0-3 | 176 | 0.30 | 0.0477 | [-0.26;0.36] | .983 | 0.0005 |
|  | 4-7 | 308 | -0.64 | -0.0725 | [-0.30;0.15] | .908 | 0.0013 |
|  | 8-10 | 344 | -0.16 | -0.0182 | [-0.24;0.20] | .990 | 0.0001 |

*Note.* The respective matched typically developing children group is used as the reference for regression coefficients and compared to mTBI at the ages 0-3, 4-7 and 8-10. Abbreviations: Df = Residual degrees of freedom, CI = confidence interval, CB = cingulum bundle, SLF = superior longitudinal fasciculus, CC = corpus callosum.

**Supplementary Table S6**

*Inferential Statistics for the Effect of Age-At-Injury on Mean Diffusivity*

| Tract | Age-at-Injury | Df | Test statistic | $\hat{\beta}$ | 95% CI | *p* | *f^2^* |
| --- | --- | --- | --- | --- | --- | --- | --- |
| CB left | | | | | | | |
|  | 0-3 | 177 | 0.83 | 0.1212 | [-0.17;0.41] | .953 | 0.0039 |
|  | 4-7 | 309 | 1.41 | 0.1558 | [-0.06;0.37] | .931 | 0.0064 |
|  | 8-10 | 354 | 0.04 | 0.0046 | [-0.21;0.22] | .967 | <0.0001 |
| CB right | | | | | | | |
|  | 0-3 | 177 | 0.93 | 0.1346 | [-0.15;0.42] | .953 | 0.0049 |
|  | 4-7 | 309 | 1.36 | 0.1551 | [-0.07;0.38] | .931 | 0.0060 |
|  | 8-10 | 354 | -0.17 | -0.0192 | [-0.24;0.20] | .967 | 0.0001 |
| SLF II left | | | | | | | |
|  | 0-3 | 176 | -0.81 | -0.1283 | [-0.44;0.19] | .953 | 0.0037 |
|  | 4-7 | 308 | -1.35 | -0.1471 | [-0.36;0.07] | .931 | 0.0059 |
|  | 8-10 | 344 | 0.84 | 0.0958 | [-0.13;0.32] | .953 | 0.0020 |
| SLF II right | | | | | | | |
|  | 0-3 | 176 | -0.58 | -0.0832 | [-0.37;0.20] | .953 | 0.0019 |
|  | 4-7 | 308 | 1.36 | 0.1396 | [-0.06;0.34] | .931 | 0.0060 |
|  | 8-10 | 344 | 0.22 | 0.0254 | [-0.21;0.26] | .967 | 0.0001 |
| SLF III left | | | | | | | |
|  | 0-3 | 176 | 0.13 | 0.0199 | [-0.29;0.33] | .967 | 0.0001 |
|  | 4-7 | 308 | 0.38 | 0.0403 | [-0.17;0.25] | .967 | 0.0005 |
|  | 8-10 | 344 | 1.03 | 0.1168 | [-0.11;0.34] | .953 | 0.0031 |
| SLF III right | | | | | | | |
|  | 0-3 | 176 | -0.80 | -0.1179 | [-0.41;0.17] | .953 | 0.0037 |
|  | 4-7 | 308 | 1.24 | 0.1306 | [-0.08;0.34] | .931 | 0.0050 |
|  | 8-10 | 344 | 0.32 | 0.0358 | [-0.19;0.26] | .967 | 0.0003 |
| CC 1 | | | | | | | |
|  | 0-3 | 176 | -0.57 | -0.0905 | [-0.40;0.22] | .953 | 0.0019 |
|  | 4-7 | 308 | 0.20 | 0.0247 | [-0.23;0.27] | .967 | 0.0001 |
|  | 8-10 | 344 | 1.50 | 0.1625 | [-0.05;0.38] | .931 | 0.0066 |
| CC 2 | | | | | | | |
|  | 0-3 | 177 | 0.13 | 0.0189 | [-0.28;0.32] | .967 | 0.0001 |
|  | 4-7 | 309 | 0.55 | 0.0638 | [-0.16;0.29] | .953 | 0.0010 |
|  | 8-10 | 354 | 0.55 | 0.0602 | [-0.15;0.27] | .953 | 0.0009 |
| CC 3 | | | | | | | |
|  | 0-3 | 176 | 1.34 | 0.2075 | [-0.10;0.51] | .931 | 0.010 |
|  | 4-7 | 308 | -0.05 | -0.0058 | [-0.23;0.20] | .967 | <0.0001 |
|  | 8-10 | 344 | 0.47 | 0.0536 | [-0.17;0.28] | .953 | 0.0007 |
| CC 4 | | | | | | | |
|  | 0-3 | 177 | 0.63 | 0.0925 | [-0.20;0.38] | .953 | 0.0022 |
|  | 4-7 | 309 | -1.25 | -0.1454 | [-0.38;0.08] | .931 | 0.0050 |
|  | 8-10 | 354 | -0.49 | -0.0501 | [-0.25;0.15] | .953 | 0.0007 |
| CC 5 | | | | | | | |
|  | 0-3 | 176 | -0.08 | -0.0126 | [-0.32;0.30] | .967 | <0.0001 |
|  | 4-7 | 308 | 0.14 | 0.0159 | [-0.21;0.24] | .967 | 0.0001 |
|  | 8-10 | 344 | 0.34 | 0.0372 | [-0.18;0.26] | .967 | 0.0003 |
| CC 6 | | | | | | | |
|  | 0-3 | 176 | 1.46 | 0.2105 | [-0.07;0.49] | .931 | 0.012 |
|  | 4-7 | 308 | 1.18 | 0.1435 | [-0.10;0.38] | .931 | 0.0045 |
|  | 8-10 | 344 | 0.52 | 0.0609 | [-0.17;0.29] | .953 | 0.0008 |
| CC 7 | | | | | | | |
|  | 0-3 | 176 | 0.92 | 0.1361 | [-0.16;0.43] | .953 | 0.0048 |
|  | 4-7 | 308 | 0.19 | 0.0222 | [-0.21;0.25] | .967 | 0.0001 |
|  | 8-10 | 344 | 0.58 | 0.0649 | [-0.16;0.29] | .953 | 0.0010 |

*Note.* The respective matched typically developing children group is used as the reference for regression coefficients and compared to mTBI at the ages 0-3, 4-7 and 8-10. Abbreviations: Df = Residual degrees of freedom, CI = confidence interval, CB = cingulum bundle, SLF = superior longitudinal fasciculus, CC = corpus callosum.
